# Supplementary material for: Early warnings of COVID-19 outbreaks across Europe from social media
Source: Sci Rep. 2021 Jan 25;11:2147. doi: 10.1038/s41598-021-81333-1 (PMC7835375; doi:10.1038/s41598-021-81333-1)
Supplement: Supplementary file 1 — Supplementary Information [file 41598_2021_81333_MOESM1_ESM.docx]

**Supplementary Information**

Early warnings of COVID-19 outbreaks across Europe from social media

Milena Lopreite, Pietro Panzarasa, Michelangelo Puliga, Massimo Riccaboni


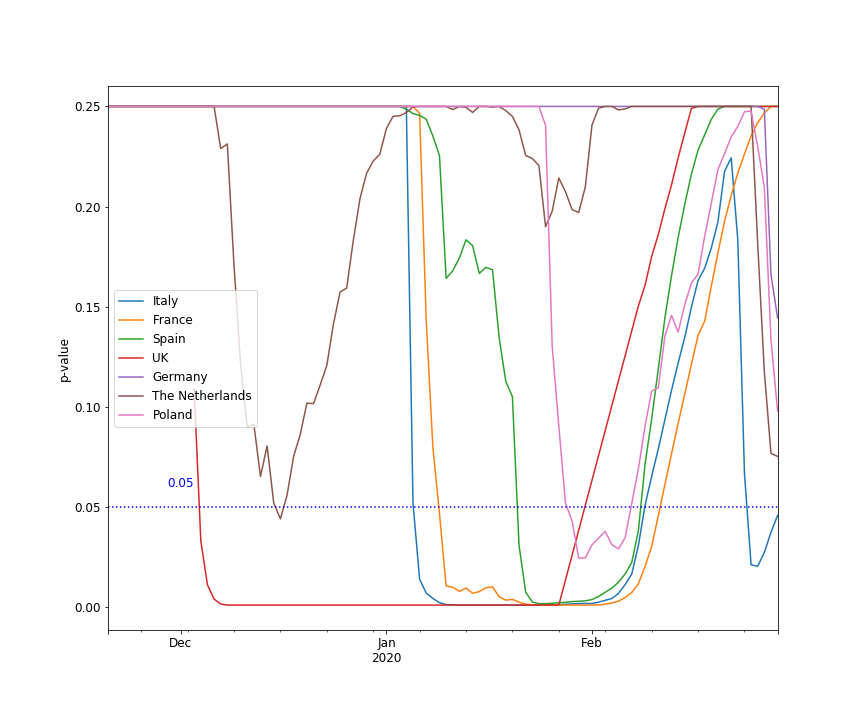


**Fig. S1 Two-sample Anderson-Darling test of the difference between cumulative distributions of number of tweets citing pneumonia and posted in two corresponding winter seasonal periods (2018-2019 and 2019-2020) for each of the 7 European countries**. The graph reports the average *p-*values over moving window widths *w* $\epsilon[50, 70]$ computed with daily frequency.


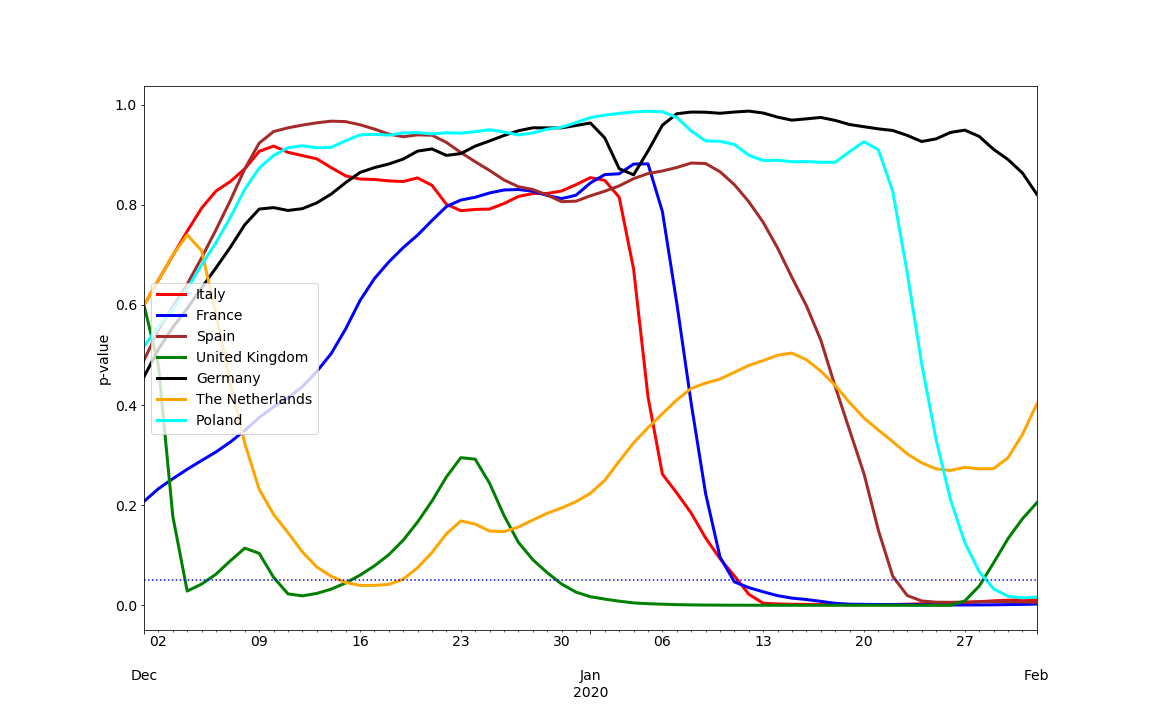


**Fig. S2 Two-sample Kolmogorov-Smirnov test of the difference between cumulative distributions of number of tweets citing pneumonia and posted in the winter seasonal period 2019-2020 and in the corresponding five preceding periods since 2014-2015 for each of the 7 European countries**. The graph reports the average of the *p-*values obtained using each of the five preceding winter seasons. In turn, the *p*-values related to each individual season are averages over moving window widths *w* $\epsilon[50, 70]$ computed with daily frequency.


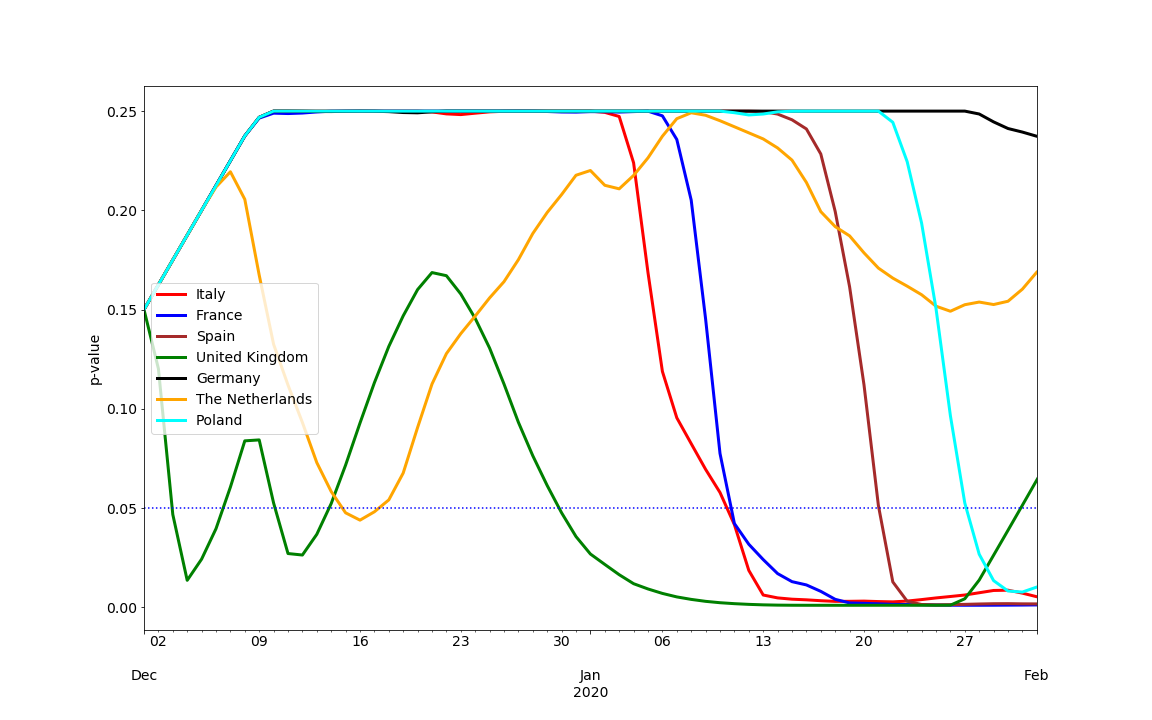


**Fig. S3 Two-sample Anderson-Darling test of the difference between cumulative distributions of number of tweets citing pneumonia and posted in the winter seasonal period 2019-2020 and in the corresponding five preceding periods since 2014-2015 for each of the 7 European countries**. The graph reports the average of the *p-*values obtained using each of the five preceding winter seasons. In turn, the *p*-values related to each individual season are averages over moving window widths *w* $\epsilon[50, 70]$ computed with daily frequency.


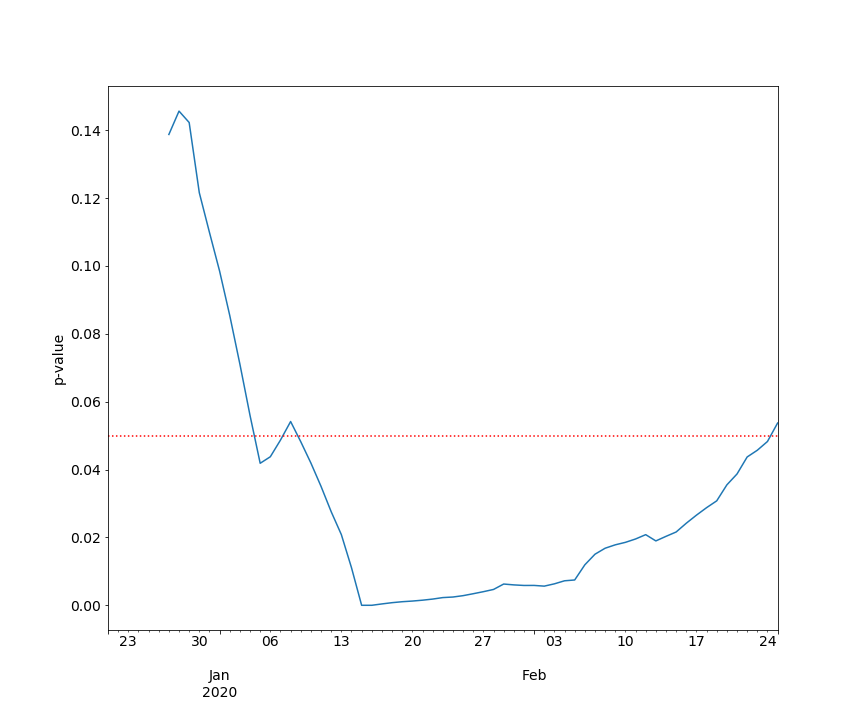


**Fig. S4 Two-sample Kolmogorov-Smirnov test of the difference between cumulative distributions of number of tweets citing dry cough and posted in two corresponding winter seasonal periods (2018-2019 and 2019-2020) in 7 European countries**. The graph reports the average *p-*values over moving window widths *w* $\epsilon[50, 70]$ computed with daily frequency.

**
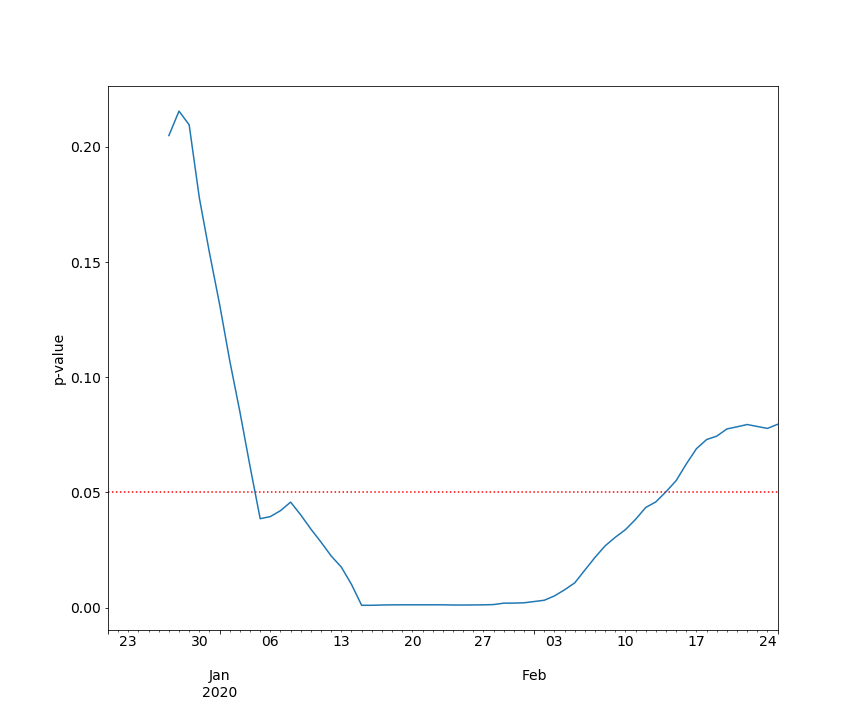
**

**Fig. S5 Two-sample Anderson-Darling test of the difference between cumulative distributions of number of tweets citing dry cough and posted in two corresponding winter seasonal periods (2018-2019 and 2019-2020) in 7 European countries**. The graph reports the average *p-*values over moving window widths *w* $\epsilon[50, 70]$ computed with daily frequency.

| **Countries** | **Periods** |
| --- | --- |
| Italy | 2020/01/06 - 2020/02/07 |
| France | 2020/01/10 - 2020/02/08 |
| Spain | 2020/01/22 - 2020/02/06 |
| UK | 2019/12/05 - 2020/01/28 |
| The Netherlands | 2019/12/15 - 2019/12/18 |
| Poland | 2020/02/05 - 2020/02/06 |

**Table S1 Countries and time periods in the 2019-2020 winter season characterized by excess posting on pneumonia according to the Kolmogorov-Smirnov tests.** In these periods the cumulative distributions of pneumonia-related tweets differ from the distributions in the corresponding time periods in the 2018-2019 season (K-S tests at the 0.05 level of significance).

| **Country** | **Period** |
| --- | --- |
| Italy | 2020/01/06 – 2020/02/29 |
| France | 2020/01/09 – 2020/02/11 |
| Spain | 2020/01/21 – 2020/02/08 |
| UK | 2019/12/04 – 2020/01/30 |
| The Netherlands | 2019/12/16 – 2019/12/16 |
| Poland | 2020/01/29 – 2020/02/06 |

**Table S2 Countries and time periods in the 2019-2020 winter season characterized by excess posting on pneumonia according to Anderson-Darling tests.** These are the periods in which the cumulative distributions of dry cough-related tweets differ from the distributions in the corresponding time periods in the 2018-2019 season (A-D tests at the 0.05 level of significance).

| **Country** | **2014-2015** | **2015-2016** | **2016-2017** | **2017-2018** | **2018-2019** |
| --- | --- | --- | --- | --- | --- |
| **France** | 0.36066  (0.00064) | 0.39344  (0.00013) | 0.36066  (0.00064) | 0.44262  (0.00001) | 0.40984  (0.00006) |
| **Germany** | 0.09836  (0.93268) | 0.06530  (0.99742) | 0.08197  (0.98783) | 0.14754  (0.52379) | 0.06557  (0.99956) |
| **Italy** | 0.34426  (0.00133) | 0.39344  (0.00013) | 0.34426  (0.00133) | 0.34426  (0.00133) | 0.45902  (0.00000) |
| **The Netherlands** | 0.26230  (0.02967) | 0.18033  (0.27603) | 0.27869  (0.01712) | 0.21173  (0.11129) | 0.22812  (0.07130) |
| **Poland** | 0.37324  (0.00036) | 0.36548  (0.00075) | 0.31064  (0.00452) | 0.27581  (0.01716) | 0.26202  (0.02566) |
| **Spain** | 0.34426  (0.00133) | 0.32787  (0.00266) | 0.32787  (0.00266) | 0.32787  (0.00266) | 0.31148  (0.00512) |
| **UK** | 0.37705  (0.00030) | 0.37705  (0.00030) | 0.42623  (0.00002) | 0.37705  (0.00030) | 0.36066  (0.00064) |

**Table S3 Two-sample Kolmogorov-Smirnov test of the difference between cumulative distributions of number of tweets citing pneumonia posted in the winter seasonal period 2019-2020 and in each of the corresponding five periods since 2014-2015 for each of the 7 European countries**. For each country and winter seasonal period, the table reports the values of the statistical test (corresponding *p*-values are shown in parentheses).

| **Country** | **2014-2015** | **2015-2016** | **2016-2017** | **2017-2018** | **2018-2019** |
| --- | --- | --- | --- | --- | --- |
| **France** | 9.39622  (0.00100) | 10.05256  0.00100) | 7.00257  (0.00100) | 14.51323  (0.00100) | 9.64127  (0.00100) |
| **Germany** | -0.82499  (0.25000) | -1.13954  (0.25000) | -1.05783  (0.25000) | 0.62221  (0.18304) | -1.09790  (0.25000) |
| **Italy** | 8.17272  (0.00100) | 11.00139  (0.00100) | 5.74394  (0.00189) | 5.05182  (0.00332) | 12.30459  (0.00100) |
| **The Netherlands** | 2.77755  (0.02381) | 1.01411  (0.12474) | 2.39161  (0.03388) | 0.52939  (0.20061) | 1.64154  (0.06830) |
| **Poland** | 8.38603  (0.00100) | 6.24365  (0.00128) | 4.13372  (0.00719) | 3.03733  (0.01883) | 3.73690  (0.01014) |
| **Spain** | 7.95646  (0.00100) | 7.28634  (0.00100) | 7.26826  (0.00100) | 6.87016  (0.00100) | 6.12392  (0.00140) |
| **UK** | 5.28041  (0.00276) | 5.36497  (0.00257) | 7.49829  (0.00100) | 5.45749  (0.00239) | 4.34225  (0.00602) |

**Table S4 Two-sample Anderson-Darling test of the difference between cumulative distributions of number of tweets citing pneumonia posted in the winter seasonal period 2019-2020 and in each of the corresponding five periods since 2014-2015 for each of the 7 European countries**. For each country and winter seasonal period, the table reports the values of the statistical test (corresponding *p*-values are shown in parentheses).

| **Country/Region** | **Users 2020** | **Users 2019** | **Relative variation**  **2020-2019** | **Absolute variation**  **2020-2019** |
| --- | --- | --- | --- | --- |
| **Germany**  Total number of tweets = 452 | | | | |
| Rheinland-Pfalz | 14 | 4 | 2.50 | 10 |
| Hessen | 28 | 9 | 2.11 | 19 |
| Baden-Württemberg* | 27 | 11 | 1.45 | 16 |
| Nordrhein-Westfalen* | 46 | 19 | 1.42 | 27 |
| Schleswig-Holstein | 14 | 6 | 1.33 | 8 |
| Hamburg | 18 | 8 | 1.25 | 10 |
| Berlin | 56 | 30 | 0.87 | 26 |
| Bayern* | 26 | 20 | 0.30 | 6 |
| Niedersachsen | 14 | 12 | 0.17 | 2 |
| Total number of users | 243 | 119 | 1.04 | 124 |
|  | | | | |
| **Spain**  Total number of tweets = 2,245 | | | | |
| Castilla-La Mancha* | 11 | 1 | 10.00 | 10 |
| Comunidad de Madrid* | 203 | 52 | 2.90 | 151 |
| Cataluña | 122 | 34 | 2.59 | 88 |
| Aragón | 11 | 4 | 1.75 | 7 |
| Extremadura | 158 | 68 | 1.32 | 90 |
| Islas Canarias | 13 | 6 | 1.17 | 7 |
| Andalucía | 83 | 42 | 0.98 | 41 |
| Galicia | 15 | 8 | 0.88 | 7 |
| Comunidad Valenciana | 38 | 24 | 0.58 | 14 |
| País Vasco | 11 | 7 | 0.57 | 4 |
| Total number of users | 665 | 246 | 1.70 | 419 |
|  | | | | |
| **France**  Total number of tweets = 2,112 | | | | |
| Provence-Alpes-Côte d'Azur | 57 | 11 | 4.18 | 46 |
| Bretagne | 24 | 6 | 3.00 | 18 |
| Centre-Val de Loire | 30 | 8 | 2.75 | 22 |
| Grand Est* | 54 | 15 | 2.60 | 39 |
| Auvergne-Rhône-Alpes | 67 | 19 | 2.53 | 48 |
| Île-de-France* | 361 | 105 | 2.44 | 256 |
| Normandie | 32 | 10 | 2.20 | 22 |
| Nouvelle-Aquitaine | 42 | 14 | 2.00 | 28 |
| Hauts-de-France | 49 | 20 | 1.45 | 29 |
| Pays de la Loire | 36 | 15 | 1.40 | 21 |
| Occitanie | 43 | 19 | 1.26 | 24 |
| Bourgogne-Franche-Comté | 21 | 12 | 0.75 | 9 |
| Total number of users | 816 | 254 | 2.21 | 562 |
|  | | | | |
| **Italy**  Total number of tweets = 1,097 | | | | |
| Friuli-Venezia Giulia | 11 | 2 | 4.50 | 9 |
| Piemonte* | 20 | 7 | 1.86 | 13 |
| Emilia-Romagna* | 19 | 7 | 1.71 | 12 |
| Umbria | 87 | 44 | 0.98 | 43 |
| Lazio | 61 | 32 | 0.91 | 29 |
| Veneto | 20 | 12 | 0.67 | 8 |
| Campania | 16 | 10 | 0.60 | 6 |
| Sicily | 19 | 12 | 0.58 | 7 |
| Toscana | 23 | 16 | 0.44 | 7 |
| Lombardia* | 201 | 151 | 0.33 | 50 |
| Total number of users | 477 | 293 | 0.63 | 184 |
|  | | | | |
| **The Netherlands**  Total number of tweets = 380 | | | | |
| Noord-Brabant* | 16 | 8 | 1.00 | 8 |
| Zuid-Holland | 33 | 17 | 0.94 | 16 |
| Gelderland | 17 | 9 | 0.89 | 8 |
| Noord-Holland | 52 | 30 | 0.73 | 22 |
| Total number of users | 118 | 64 | 0.84 | 54 |
|  | | | | |
| **Poland**  Total number of tweets *=* 244 | | | | |
| Mazowieckie* | 25 | 10 | 1.50 | 15 |
| Łódzkie | 31 | 17 | 0.82 | 14 |
| Total number of users | 56 | 27 | 1.07 | 29 |
|  | | | | |
| **United Kingdom**  Total number of tweets = 4,451 | | | | |
| England* | 1,462 | 484 | 2.02 | 978 |
| Wales* | 66 | 22 | 2.00 | 44 |
| Northern Ireland | 36 | 14 | 1.57 | 22 |
| Scotland | 192 | 83 | 1.31 | 109 |
| Total number of users | 1,756 | 603 | 1.91 | 1,153 |

**Table S5 European regions associated with an excess number of unique users discussing pneumonia, after filtering out press releases and news accounts**. Highlighted in red are the regions that, based on the Wikipedia pages that summarize the statistics on COVID-19 per each country, reported active local cases of COVID-19 in the initial period between 15 February and 7 March 2020. Marked with a star (*) are the regions with the highest number of COVID-19 cases per capita (Source: Wikipedia, 20 April 2020).

| **European region** | **Number of unique users** |
| --- | --- |
| England | 96 |
| Comunidad de Madrid | 35 |
| Île-de-France | 27 |
| Centre-Val de Loire | 26 |
| Andalucía | 23 |
| Cataluña | 17 |
| Comunidad Valenciana | 14 |
| Hauts-de-France | 8 |
| Lombardia | 8 |
| Umbria | 7 |
| Thüringen | 6 |
| Pays de la Loire | 6 |
| Auvergne-Rhône-Alpes | 6 |
| Occitanie | 6 |
| Región de Murcia | 5 |
| Emilia-Romagna | 5 |
| Provence-Alpes-Côte d'Azur | 4 |
| Castilla-La Mancha | 4 |
| Scotland | 3 |
| Noord-Holland | 3 |
| Aragón | 3 |
| Łódzkie | 3 |
| Castilla y León | 3 |
| Gelderland | 3 |
| Berlin | 3 |
| Islas Baleares | 2 |
| Wales | 2 |
| Overijssel | 2 |
| Nouvelle-Aquitaine | 2 |
| Utrecht | 2 |
| Veneto | 2 |
| Friuli-Venezia Giulia | 2 |
| Zuid-Holland | 2 |
| Galicia | 2 |
| Grand Est | 2 |
| Campania | 2 |
| Bretagne | 1 |
| Baden-Württemberg | 1 |
| Trentino-Alto Adige | 1 |
| Toscana | 1 |
| Groningen | 1 |
| Cantabria | 1 |
| Hamburg | 1 |
| Extremadura | 1 |
| Northern Ireland | 1 |
| Normandie | 1 |
| Nordrhein-Westfalen | 1 |
| Niedersachsen | 1 |
| Kujawsko-Pomorskie | 1 |
| Ceuta y Melilla | 1 |

**Table S6 European regions and number of unique users discussing dry cough between 1 December 2019 and 30 January 2020.** Usual adjustments have been made to filter out messages related to press releases and news account.
